# Tracing Tomorrow: young people’s preferences and values related to use of personal sensing to predict mental health, using a digital game methodology

**DOI:** 10.1136/bmjment-2023-300897

**Published:** 2024-03-20

**Authors:** Gabriela Pavarini, David M Lyreskog, Danielle Newby, Jessica Lorimer, Vanessa Bennett, Edward Jacobs, Laura Winchester, Alejo Nevado-Holgado, Ilina Singh

**Affiliations:** 1 Ethox Centre, Oxford Population Health, University of Oxford, Oxford, UK; 2 Wellcome Centre for Ethics and Humanities, University of Oxford, Oxford, UK; 3 Department of Psychiatry, University of Oxford, Oxford, UK; 4 Centre for Statistics in Medicine, Nuffield Department of Orthopaedics, Rheumatology, and Musculoskeletal Sciences, University of Oxford, Oxford, UK

**Keywords:** Child & adolescent psychiatry

## Abstract

**Background:**

Use of personal sensing to predict mental health risk has sparked interest in adolescent psychiatry, offering a potential tool for targeted early intervention.

**Objectives:**

We investigated the preferences and values of UK adolescents with regard to use of digital sensing information, including social media and internet searching behaviour. We also investigated the impact of risk information on adolescents’ self-understanding.

**Methods:**

Following a Design Bioethics approach, we created and disseminated a purpose-built digital game (www.tracingtomorrow.org) that immersed the player-character in a fictional scenario in which they received a risk assessment for depression Data were collected through game choices across relevant scenarios, with decision-making supported through clickable information points.

**Findings:**

The game was played by 7337 UK adolescents aged 16–18 years. Most participants were willing to personally communicate mental health risk information to their parents or best friend. The acceptability of school involvement in risk predictions based on digital traces was mixed, due mainly to privacy concerns. Most participants indicated that risk information could negatively impact their academic self-understanding. Participants overwhelmingly preferred individual face-to-face over digital options for support.

**Conclusions:**

The potential of digital phenotyping in supporting early intervention in mental health can only be fulfilled if data are collected, communicated and actioned in ways that are trustworthy, relevant and acceptable to young people.

**Clinical implications:**

To minimise the risk of ethical harms in real-world applications of preventive psychiatric technologies, it is essential to investigate young people’s values and preferences as part of design and implementation processes.

**WHAT IS ALREADY KNOWN ON THIS TOPIC**

- There is substantive literature on the communication of health risk information following clinical and commercial genetic testing, but we need more understanding of appropriate support for young people in the context of psychiatric risk identified from naturalistic digital data such as social media uploads and views, communication patterns and internet searches.

**WHAT THIS STUDY ADDS**

- This study is the first to employ a ‘Design Bioethics’ approach, investigating young people’s ethically relevant preferences at scale through a digital game. Game choices suggested that most adolescents would disclose risk information to a close other and would accept support (preferably in face-to-face, one-to-one formats). However, they also revealed privacy concerns regarding sharing data and results and a potential negative impact of risk information for adolescents’ self-understanding.

**HOW THIS STUDY MIGHT AFFECT RESEARCH, PRACTICE OR POLICY**

- Young people’s need for transparency, control and support in the context of personal sensing must be considered in research design and policy implementation of personal and behavioural sensing applications.

## Introduction

The ability to collect digital data on human behaviour expands every year. Diverse activities are tracked, from text messages to footsteps, providing unique opportunities for prediction of human behaviour.^1^ Our digital traces also provide opportunities for early detection of mental health difficulties.^2^ Recent computational studies suggest that onset of depression and post-traumatic stress disorder can be predicted from social media posts prior to diagnosis,^3 4^ sparking growing interest in algorithmic analysis of digital traces as a front-line tool for early intervention in adolescent psychiatry.^5 6^ Among many benefits is the fact that digital footprints are passively collected and widely available, so screening is less costly and burdensome than clinical or survey-based assessments, and can help identify difficulties among those who might be reluctant to seek support.^7^ The potential population health benefits of large-scale cross-sector data sharing and linkage (eg, between schools and health systems) have also been highlighted, including enhanced efficiency and service integration.^8 9^ The wide-ranging surveillance capabilities attributed to a growing array of digital tools, many embedded in everyday devices such as mobile phones, have been the subject of increasing ethical scrutiny. ‘Personal sensing’ is a term proposed to increase transparency about the potentially intrusive objectives of the use of these tools, as it ‘conveys the intent and practice, as well as the personal nature of the behaviours and states we [researchers] are attempting to detect’ (Mohr *et al*, 2020).

Although the research on early detection of mental health risk from digital data is still developing, implementation seems to be progressing quickly. Services that claim to detect emotional risk from digital footprints are already available (eg, Smoothwall, Steer, Social Sentinel), targeting schools, parents and young people. Social media platforms have integrated systems to detect mental health risk by screening users’ posts.^10^ Implementation of such services must not be guided only by scientific evidence; robust ethical analysis must be included throughout the process. Crucially, such analysis must integrate the perspectives of young people themselves, which requires systematic efforts to ensure that these perspectives are informed, balanced and reliable. Without such efforts, services are likely to fail. For instance, the Samaritans Radar, a Twitter plug-in launched in 2014 enabling users to monitor each other’s mental health, was suspended 9 days after its launch due to public backlash around privacy concerns.^11^

While a young person might not perceive some aspects of personal sensing (eg, Instagram posts) as private information, mental health insights arising from algorithmic analysis of digital traces are highly sensitive. Personal mental health information can attract discrimination, influence criminal justice proceedings or lead to coercive interventions.^12^ The use and processing of this information by schools and technology firms such as social media companies therefore raise concerns with regard to informed consent and data privacy.^13 14^ This includes challenges to *constitutional* privacy (eg, interference from general practitioner (GP), social media platforms, friends or school staff) and *informational* privacy (eg, control over mental health risk data collection and dissemination).^14^

A further ethical issue concerns relative risks and benefits of risk information itself. Previous research suggests that information about (genetic) susceptibility to depression in the absence of psychoeducation increases symptom reporting and decreases confidence in coping skills.^15^ Risk information might impact a young person’s self-understanding across different domains, including (academic) performance and moral agency. These perceptions are tied to values of identity: an understanding of oneself as a capable and robust agent, and someone able to help and support others.^16^

The potential for personal sensing to improve young people’s mental health outcomes also depends on how risk information is communicated, and the support offered to those identified as ‘at risk’. Several factors need to be considered, including how risk information is delivered and received between parties (eg, a young person and their doctor); to whom young people disclose risk information; and support options sought by and offered following disclosure. Even with support, young people are unlikely to benefit if support sources are perceived as irrelevant, inaccessible or stigmatising.

Given the pace and scale of healthcare advances driven by digital and artificial intelligence (AI) capabilities, we need tools to understand whether and how such advances intersect with young people’s values and priorities. Ideally, we would be able to conduct relevant research efficiently and at scale, in order to rapidly assess attitudes and perspectives from diverse groups of young people. Meaningful engagement requires enabling adolescents to imagine and to consider complex healthcare scenarios and their potential implications.

In response to these challenges, we developed a Design Bioethics approach,^17^ which entails the iterative design and use of purpose-built, technology-driven tools for bioethics research and engagement. Working with young people and game developers, we designed Tracing Tomorrow, a digital narrative game that transports young people into a realistic predictive psychiatric scenario.

The game focuses on depression, which typically has its onset in adolescence and has been considered ‘a prime target for early intervention’.^18^ We designed choice situations that engage moral values that have been identified as relevant to digital phenotyping, including privacy,^19–21^ identity—particularly self-understanding^22^ and autonomy.^20 23^ Through game choices, we investigate young people’s preferences centred around sources of support and trust, sources of information, and data control.

## Objectives

This study aimed to investigate young people’s preferences and values related to the use of personal sensing, particularly social media uploads and views, and internet searching, for early detection of risk of mental health challenges. We report Tracing Tomorrow findings from a large sample of UK adolescents, focusing on four key research questions:

- Who—if anyone—do young people trust with information about risk of poor mental health?
- What are young people’s preferred sources of information and support?
- What are young people’s normative dispositions towards data sharing and communication in this context?
- Does risk information affect a young person’s self-understanding?

Given previously documented gender differences in help-seeking, stigma and mental health knowledge,^24^ we also report exploratory analyses investigating whether young people’s preferences are related to gender.

## Methods

### Game co-design

Tracing Tomorrow was developed with extensive input from UK adolescents. The focus on depression and digital footprints was decided based on a consultation with a representative sample of 751 UK adolescents aged 16–18 years. Core ethical themes, narrative focus and game style were based on in-person qualitative consultations with 34 UK adolescents in the same age range. The NeurOX Young People’s Advisory Group (NeurOX YPAG), a cohort of 42 adolescents aged 14–18 years across Oxfordshire, supported design across all stages, including feedback on the narrative, outcome options, language, user experience, visual style and title. Their support helped increase the game’s relevance, inclusivity and accessibility and minimise the risk of emotional triggering. Three NeurOX YPAG members critically reviewed this paper. Further details about co-design and consultations are provided elsewhere.^25^ The final version of Tracing Tomorrow was tested in a validation study, showing that the game generated higher presence, engagement and insight in UK adolescents, than an equivalent, vignette survey.^26^

### Recruitment

The Tracing Tomorrow game was made freely available at www.tracingtomorrow.org. The game was disseminated via social media influencers, Instagram adverts and a radio show. Influencers were chosen based on size of following, audience age and intersectional representation. The campaign took place between 30 January and 1 March 2020. Any individual could access the game; however, the current study only included data from UK residents aged 16–18 years old, who responded to demographic questions and at least the first question during gameplay. We did not predetermine a sample size.

Prior to gameplay, a screen informed participants that they would be participating anonymously in research led by the University of Oxford.

### Tracing Tomorrow game

Before starting the game, participants were asked their age, gender (female, male, other or prefer not to say) and place of residence (England, Scotland, Wales, Northern Ireland or ‘outside UK’). To encourage authentic decision-making, the narrative was presented in the first person and participants were asked to ‘make choices as yourself; complete your journey your way’. An initial question was used to enhance personalisation (eg, choosing a style of clothes to wear). No avatar was used to represent the player. All characters were represented as shadows to enhance identification across demographics.

Tracing Tomorrow is set in the weeks leading up to the player-character’s final school examinations. The narrative centres around communications to the player-character that they may be at risk of depression. The game leads participants to navigate several scenarios through a continuous narrative, exploring implications of receiving such a risk assessment. Each scenario culminates in a decision point, where players are asked to click on one of four outcomes to drive the narrative forward. After the choice, brief text is displayed outlining what happened next from a narrative point of view (online supplemental figure 1). A test question (Q1) is used to familiarise the player with the game structure, followed by 10 scenarios leading to relevant decision points (Q2–Q11), presented to all players in the same order. Scenarios and items (see table 1) were designed to cover the following core themes:

10.1136/bmjment-2023-300897.supp1 Supplementary data

1. *Trust to disclose*. Participants are asked to indicate who—if anyone—they trust to disclose information about risk of poor mental health and their personal experience of being monitored and notified. This is investigated in an individual scenario and in a peer group scenario.
2. *Knowledge and support*. Participants are asked to indicate (1) whether and where they would seek information to understand results from a mental health risk assessment, (2) their preferences regarding different support sources from school, and (3) whether they would accept support from peers.
3. *Normative disposition*. Participants are asked to indicate (1) whether they would sign up to a digital phenotyping programme offered by an educational institution; (2) whether they would continue an automated mental health screening offered by a social media platform, and (3) their stance on digital phenotyping results being communicated between healthcare systems and schools. The choice alternatives were designed to reflect adolescents’ preferences in relation to the value of privacy and data control.
4. *Self-understanding*. Across two dilemmas, participants indicate whether information about mental health risk could affect their self-understanding in the competence domain (ability to perform well on final examinations) and in the moral domain (willingness to help a friend going through a similar problem).

**Table 1:** Tracing Tomorrow themes, scenarios and game choices

Theme and scenario	Choices
*Information and support–information-seeking preference (Q2*) ‘Do you try to find out more about the letter (from the player’s GP, reporting their depression risk)?’	Nope. The letter is going in a drawer. I’ll Google it. I’ll call my GP. I’ll call the mental health helpline.
*Trust to disclose–disclosure target preference (Q3*) ‘(…) You wonder if you should tell someone about (the letter and your risk status). What do you do?’	I’ll take a photo of the letter and Snapchat it. I’ll talk to my parents. I’ll message my best friend. I’ll keep it to myself, thanks. This stuff is personal.
*Normative disposition–predictive service by social media platform (Q4*) ‘Which do you choose?’ (following a social media mental health notification suggesting increased risk of depression)	I’ll keep my settings the same. These notifications might be helpful. I’ll stop all notifications, delete my history and say no to tracked data. I’ll stop mental health notifications. They won’t help me. I’ll give them more info to improve my mental health notifications.
*Trust to disclose–willingness to disclose in peer group (Q5*) What do you say in response? (when a conversation among peers turns to the topic of whether it is easy to detect if someone has poor mental health)	Good question. I want to know more too. Actually…I got this letter the other day. So, I’m going to get a drink. Be right back. I’ve heard it’s possible to know some stuff by looking at people’s data.
*Normative disposition–data sharing between school and health service (Q6*) (Upon learning the GP has disclosed the player’s mental health risk results to their school) This makes you think…	Maybe it’s a good thing that the GP has told the school nurse. Maybe they could help. If I’m not actually depressed, the school shouldn’t be notified. Whatever happened to doctor–patient confidentiality? No way the school should have been told. OK, that’s good, I guess, but I hope only the school nurse knows.
*Self-understanding–academic competence self-understanding (Q7*) (When reflecting that the depression risk prediction may be accurate) Your next thought?	No. This won’t make any difference to my results. Maybe I just have to work harder to pass now. I guess knowing this could help me prepare for my examinations. I could literally fail all of my examinations because of this.
*Self-understanding–moral self-understanding (Q8*) If (a friend) got the same letter…	…I should stay out of it. I’ve got my own issues. …It’s personal. None of my business. …I reckon I can help. I know what they’re going through. …I should help. They’re my friend, and friends should help each other.
*Information and support–school support preference (Q9*) How do you respond (to an open offer from a teacher to connect player to support for mental health challenges)?	Is there a mental health app or something I could get? Thanks, but I don’t want school involved in my life like that. Maybe I could join a support group or something? Is there someone I can speak to one-to-one about it?
*Information and support–willingness to accept peer support (Q10*) (When friends offer help) Will you tell them how you feel?	No. I don’t want to be that person who’s always dumping on their friends. Yes. I really need some help right now. Yes. They know me better than anyone else. No. It’ll just make things worse.
*Normative disposition–predictive service by school (Q11*) What will you do? (a new opportunity to sign up to a personal sensing service offered by an educational institution)	Accept Reject

GP, general practitioner.

To support young people’s capacity for informed decision-making, the game includes six clickable ‘fact’ icons that provide the player with further information about mental health and digital phenotyping (online supplemental table 1). For example, one such fact states: ‘Internet or mobile tech can be used to collect or monitor signs of mental well-being in someone’. After completing the game, participants are presented with links to more information about the project and sources of mental health support. Participants were allowed to play the game more than once, but answers from duplicate internet protocol addresses were only saved once (first entry).

### Data analysis

The compareGroups package^27^ was used to create statistics stratified by gender for respondents to each game question. For each question, percentages were calculated after excluding missing values (due to dropouts throughout the game). Χ^2^ or Fisher’s exact test was used to calculate significance of the difference between gender groups. A multinomial regression (or a binomial regression model for the final question only) was used to assess the OR between each choice with gender groups (reference: female, male, did not say, other) adjusting for age and nation of residence. Analyses were conducted using R V.4.02.

## Findings

### Participants

A total of 18 932 people played the Tracing Tomorrow game between 30 January and 1 March 2020. We excluded individuals not based in the UK (n=4268), not aged 16–18 years (n=4070), those who only reported demographics (n=1286) or did not answer the first question (n=1979) resulting in a final sample of 7337. Most included participants were female, aged 16 years and based in England (table 2).

**Table 2:** Participants’ characteristics

	Female (n=5214)	Male (n=1947)	Other (n=103)	Did not say (n=73)	All (n=7337)	P value
**Age**						
16 years old	2008 (38.5%)	882 (45.3%)	42 (40.8%)	34 (46.6%)	2966 (40.4%)	<0.001
17 years old	2002 (38.4%)	729 (37.4%)	41 (39.8%)	27 (37.0%)	2799 (38.1%)	
18 years old	1204 (23.1%)	336 (17.3%)	20 (19.4%)	12 (16.4%)	1572 (21.4%)	
**Location**						
England	4505 (86.4%)	1729 (88.8%)	93 (90.3%)	68 (93.2%)	6395 (87.2%)	0.096
Northern Ireland	123 (2.36%)	41 (2.11%)	0 (0.00%)	0 (0.00%)	164 (2.24%)	
Scotland	339 (6.50%)	113 (5.80%)	6 (5.83%)	3 (4.11%)	461 (6.28%)	
Wales	247 (4.74%)	64 (3.29%)	4 (3.88%)	2 (2.74%)	317 (4.32%)	

From the final sample of 7337, the overall dropout rate from the test question (Q1) to Q11 was 31.2% (n=2292), with 4.4% of participants (n=325) dropping out after Q1. Dropout was gradual throughout the game and not linked to a particular question/scene (online supplemental figure 2). Dropout rates did not differ between age or location, but differed by gender: dropout rates for males and those who answered ‘prefer not to say’ were higher (~37%). The full dropout rates by age, gender and nation are provided in online supplemental table 2.

### Findings

#### Trust to disclose

**Figure 1:**
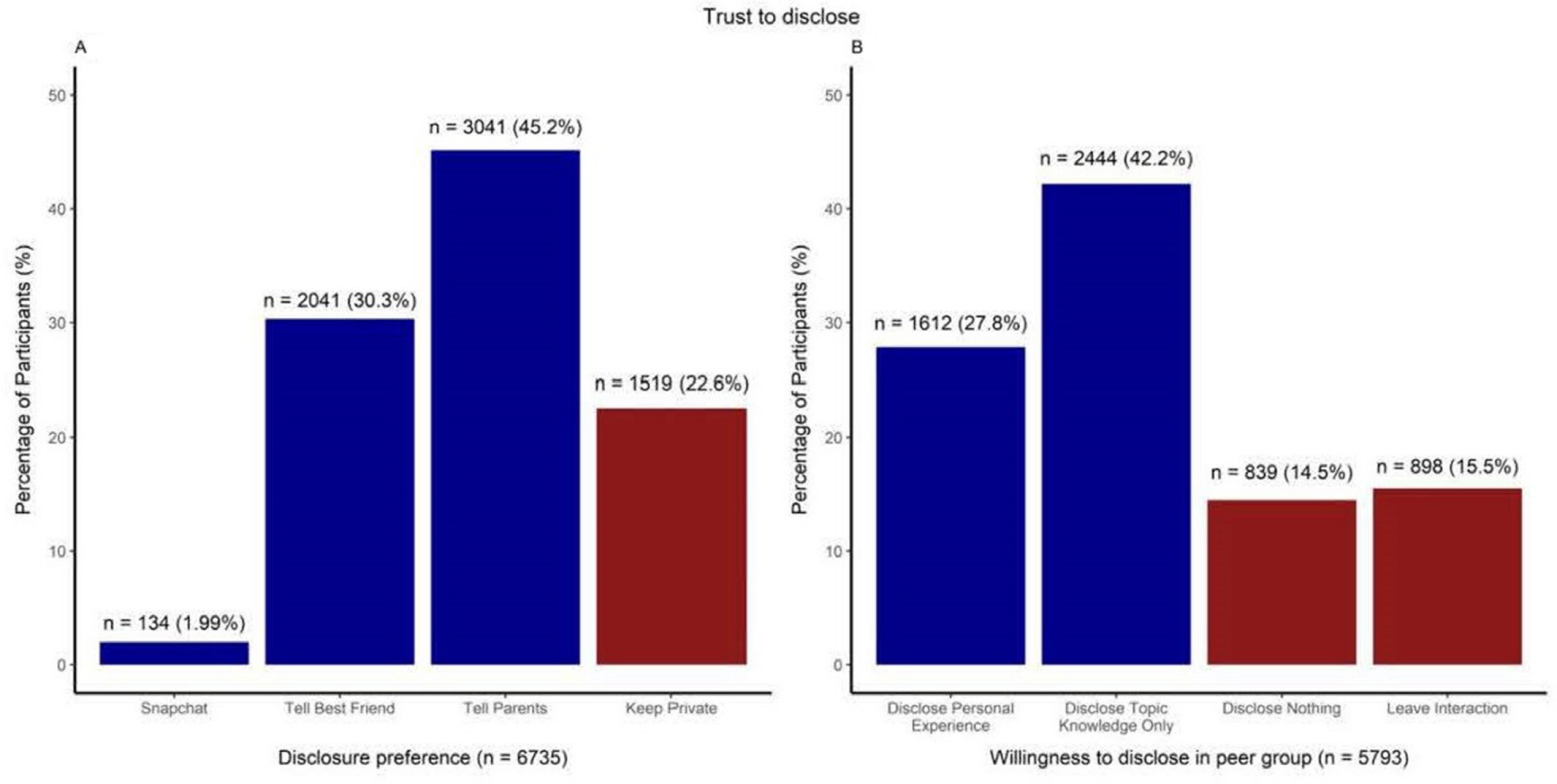
Adolescents’ disclosure target preference and willingness to disclose mental health risk status with peer group.

Most participants (75·5%) were willing to disclose mental health risk information to either their parents or best friend, 2.0% would disclose on social media (Snapchat) and 22.6% would not disclose (figure 1A). Seventy per cent were willing to talk about predicting mental health from digital data in a peer group interaction. However, participants were more willing to disclose factual knowledge about digital tracking than knowledge about their personal experiences. Thirty per cent of participants chose not to engage in any type of disclosure (figure 1B).

#### Knowledge and support

**Figure 2:**
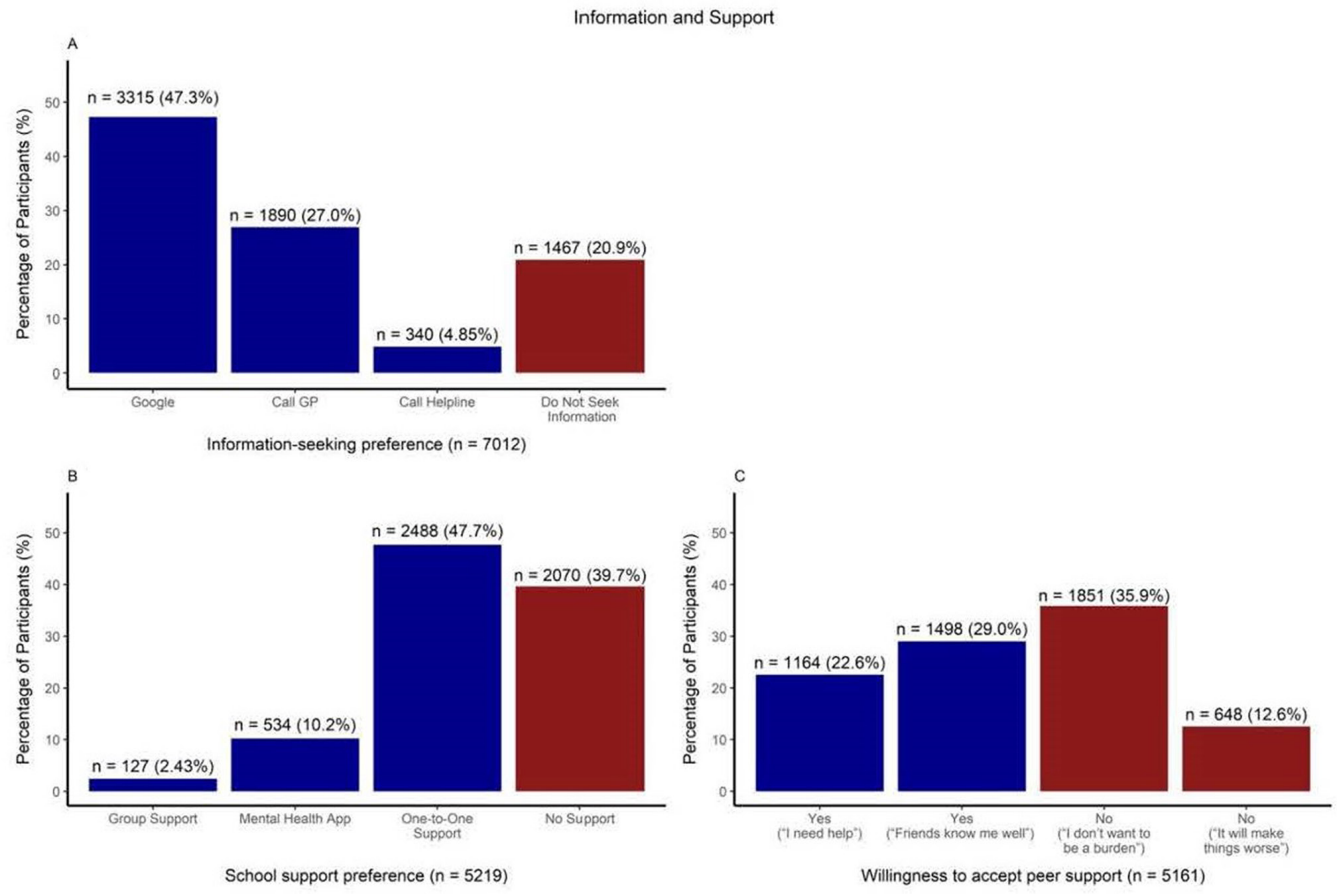
Adolescents’ information and help-seeking preferences following disclosure of mental health risk status.

About half of the participants (47.3%) would search Google following disclosure of mental health risk status, and 27% preferred to contact a medical professional. One in five would not seek further information (figure 2A). With regard to help-seeking preferences, 47.7% preferred one-to-one, face-to-face support, representing 79.1% of those participants willing to accept mental health support from their school (60.3%) (figure 2B). A minority preferred apps or school-led group support. In terms of peer support, about half of the participants (51.6%) were willing to accept support from peers; among those unwilling, the reluctance was largely due to ‘fear of being a burden’ (figure 2C).

#### Normative disposition

**Figure 3:**
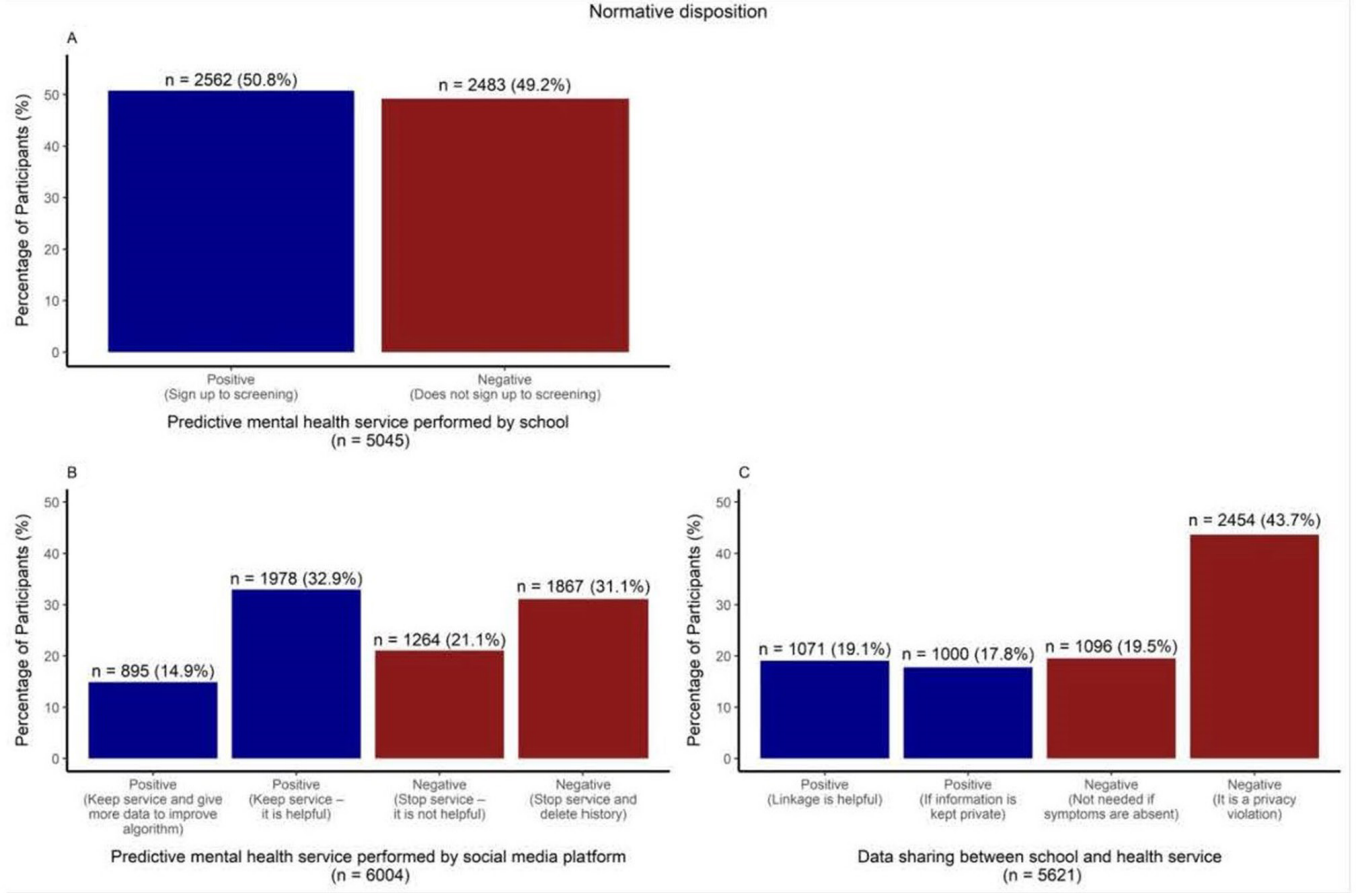
Adolescents’ normative disposition with regard to personal sensing services performed by school (A), social media platform (B) and data sharing between schools and health services (C).

About half of the participants were willing to sign up to a mental health tracking service based on digital footprints, regardless of whether this screening was performed by an educational institution (figure 3A) or a social media platform (figure 3B). When this screening was performed by a social media platform, about one-third (31.1%) chose to delete their history from the platform, and only 14.9% would share further data to improve predictions (figure 3B).

As illustrated in figure 3C, most participants did not find it acceptable for risk information to be shared between the GP and the school without prior consultation: 43.7% considered such sharing to be a privacy violation, and about one-fifth (19.5%) thought risk information should not be disclosed across systems if no symptoms are present. The remaining participants (36.8%) expressed positive attitudes to data sharing; of those, about half wanted risk information to be available only to health staff within the school (such as a nurse).

#### Self-understanding

**Figure 4:**
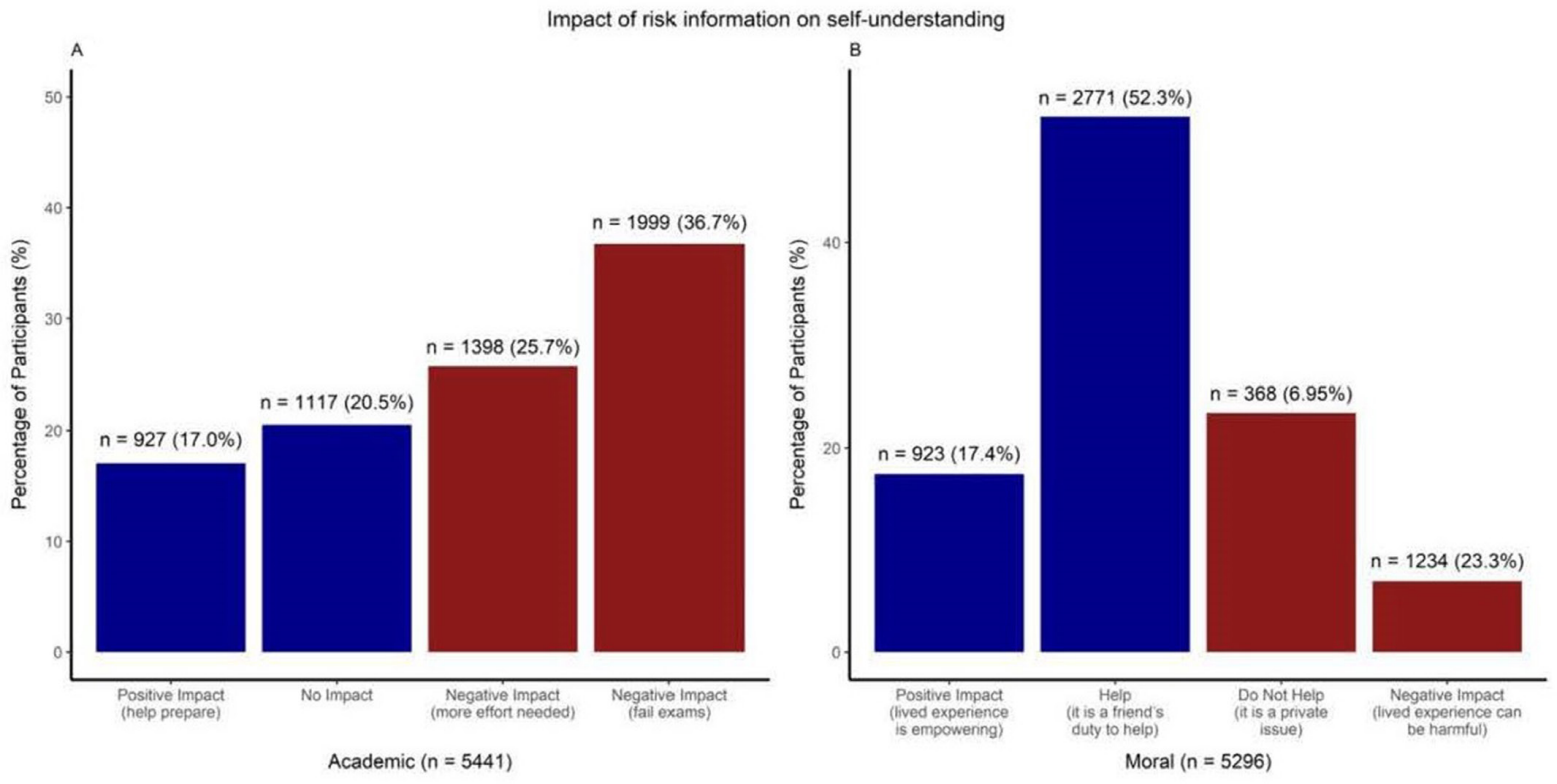
Adolescents’ perceived impact of mental health risk information on self-understanding in the academic competence domain (A) and the moral domain (B).

For 62.4% of the participants, being told they were at risk of depression negatively affected their perceived competence to perform academically. In contrast, 17% thought that knowing their risk status could help them prepare (figure 4A). The impact on moral self-understanding was less pronounced: 69.7% of participants still felt able to help a peer who was undergoing a similar challenge (ie, received information about depression risk); 17.4% considered that their lived experience empowered them to help others more effectively (figure 4B).

### Gender differences

Similar response patterns were observed across gender categories with a few notable differences (see online supplemental table 3 for descriptive statistics and online supplemental table 4 for ORs, adjusted for age and nation of residence). Compared with women/girls, men/boys were less likely disclose mental health risk status to close others but more likely to disclose it publicly on Snapchat or within a peer group (online supplemental figure 3A,B). Men/boys were less likely to seek additional information (online supplemental figure 4A) or accept school support (online supplemental figure 4B). They were also less likely to accept peer support, for fear of ‘making things worse’ (online supplemental figure 4C). For participants who identified with another gender category or did not disclose gender, CIs were too wide and overlapping to draw firm conclusions.

Men/boys showed greater acceptance of digital phenotyping by schools (online supplemental figure 5A) and social media platforms (online supplemental figure 5B), as well as of communication of results between the GP and schools (online supplemental figure 5C), compared with women/girls. Participants with non-binary gender identities exhibited high variability in responses, but generally expressed stronger privacy concerns regarding school–GP’s data sharing (online supplemental figure 5C). Participants with undisclosed gender were more likely to oppose mental health social media tracking (online supplemental figure 5B). Men/boys were less likely to indicate that risk information could affect their academic self-understanding (online supplemental figure 6A), but were more likely to indicate that it could affect their moral self-understanding, compared with women/girls (online supplemental figure 6B).

## Conclusions

Using a Design Bioethics approach,^17^ this study investigated UK adolescents’ values and preferences regarding use of personal sensing data, specifically social media views and uploads, internet searches, and use of electronic academic and clinical records, to predict risk of mental health challenges, using a co-designed digital game. Most participants were willing to personally communicate risk information to their parents or best friend, but expressed privacy concerns when results were shared between GP and school without prior consultation. Similar concerns emerged when an unsolicited ‘mental health risk notification’ revealed tracking and monitoring via social media. Even though the internet was the participants’ preferred source of *information* in response to receiving a risk prediction, face-to-face (one-to-one) meetings were overwhelmingly preferred over online options for *support* following receipt of risk information. Finally, most participants indicated that receiving depression risk information could affect their academic self-understanding.

Participants’ risk disclosure preferences impact help-seeking, particularly when clinical intervention is needed. Previous studies on mental health help-seeking suggest that parents, who were participants’ preferred confidants, typically offer an effective route for formal support.^28^ However, feedback from the NeurOX YPAG suggests that parents sometimes ‘dismiss’ mental health concerns as ‘merely teen angst’ and questioned parents’ suitability as the primary source of help, especially for early symptoms. This feedback, combined with our finding that over one-fifth of participants would not disclose results at all, highlights the need for appropriate signposting to support following mental health risk disclosure.

Even with available support, many young people may decline help. In our study, nearly half declined peer support and about two-fifths declined school support. Men/boys resisted more, consistent with previous evidence on gender differences in help-seeking.^24^ Notably, despite the rise of mental health apps,^29^ and UK National Health Service endorsement, adolescents in our study strongly preferred one-to-one, in-person support over apps. To make digital tracking a viable early intervention for mental health, accessible and acceptable support options for adolescents are crucial. Understanding their concerns regarding formal and informal support is paramount for responsible progress in the field.

Two ethically relevant considerations in school-based digital monitoring of mental health include tracking students’ digital footprints such as social media and internet searches, and sharing individual risk information with health systems.^8 14^ Our participants, particularly females and those identifying as ‘other’ gender, expressed significant privacy concerns in both areas, consistent with previous research on online privacy.^30^ To address these concerns, students should have the opportunity to provide explicit consent to tracking, monitoring and data sharing, and to opt out or withdraw from these schemes without any repercussions. Our study highlights adolescents’ concerns about data control and privacy in mental health contexts. Neglecting this principle in prevention strategies may erode trust in digital phenotyping efforts, leading to knock-on consequences,^31^ including resistance, alienation and rejection of preventive mental health approaches.

Participants’ concerns that information about risk of depression could negatively impact their academic performance suggest that, when offered without psychoeducation or other support, risk information might be harmful to young people. This was particularly true for female participants, who typically show lower positive academic self-concept.^32^ While this impact needs to be assessed experimentally, it points to the importance of informing adolescents about predictive model accuracy and malleability of effects, while ensuring access to support sources. These elements should be integrated into support strategies when returning results from personal sensing initiatives.

### Methodological considerations and future directions

This study is the first to employ a ‘Design Bioethics’ approach.^17^ In contrast to more ‘traditional’ methods in bioethics such as surveys or interviews, Tracing Tomorrow engaged young people through immersive and realistic scenarios. The game also allowed us to gauge adolescents’ perspectives rapidly and at scale. But this approach is not without limitations. The game is a simulation, so choices could be biased and not reflect ‘real-world’ preferences. We made efforts to circumvent these limitations, such as by not using avatars and explicitly encouraging participants to ‘make choices as yourself’. We also validated the approach in a separate study, where most participants indicated that their Tracing Tomorrow game choices were an authentic representation of real-life choices.^26^ Nevertheless, future studies should expand our findings by investigating preferences using ‘real-world’ data, for instance, in service settings.

The game approach also meant that the results were limited by the specific scenarios and options provided. These were based on the priorities and concerns identified during co-design with young people, as well as gaps in the literature. In a time of increased focus-predictive psychiatry using digital data harvested from non-clinical settings, where standard medical ethics principles do not explicitly govern processes of data collection, analysis, sharing or disclosure, it became important to understand the appropriate support required for young people in these contexts. Our scenarios predominantly focused on social media and internet searches, areas targeted by various screening services and notably data sources young people exhibit reluctance to share.^19^ Consequently, it is important to investigate whether the results extend to other data sources (eg, biometrics or activity levels) and environments (eg, health clinics).

Tracing Tomorrow was widely advertised, and our campaign focused on reaching minoritised groups. However, given our novel game approach, we did not explicitly recruit a representative sample and collected minimal demographics. Most participants were female, and the rate of dropout was higher among males, potentially biasing the main results. Future research should seek to test and extend our findings, focusing on understanding the specific concerns of groups who may be particularly at risk of harm from digital monitoring and surveillance, such as racial and ethnic minorities, and young people who identify as lesbian, gay, bisexual, transgender and/or queer. Similarly, it would be important to extend this research to other countries such as the USA and South Korea, where social media screening is gaining rapid popularity.^33 34^ Critically, future research should investigate whether young people’s attitudes have shifted since the COVID-19 pandemic, given the increased reliance on digital mental health tools^35^ and popularity of social media platforms.^36^ Finally, it will be interesting to ascertain how services and preferences may shift in light of new data protection legislation introduced to the UK in 2023, which substantially liberalise automated decision-making.^37^

### Clinical implications

Results from this study emphasise the need for clear and acceptable pathways to early intervention. An adolescent’s benefit from mental health risk information depends on how information is communicated, with whom it is shared, and how adolescents are supported in decisions to seek help and to disclose their risk status. Without appropriate support pathways, personal sensing efforts might violate young people’s privacy or negatively affect their self-understanding.

As digital and AI technologies encourage novel preventive efforts in child and adolescent mental health, we must be sure, not just that the benefits of these efforts outweigh the risks, but that we properly engage young people in the design, testing and implementation of our strategies. Otherwise, our efforts are likely to be resisted, and they will be more likely to fail.

## Data Availability

The Tracing Tomorrow dataset and associated data dictionary are available on the Open Science Framework (https://tinyurl.com/tracingtomorrow). Game screenshots, list of 'fact' icons, dropout data and results stratified by gender are provided in supplemental information.
