# Supplementary material for: Tracing Tomorrow: young people’s preferences and values related to use of personal sensing to predict mental health, using a digital game methodology
Source: BMJ Ment Health. 2024 Mar 20;27(1):e300897. doi: 10.1136/bmjment-2023-300897 (PMC11021752; doi:10.1136/bmjment-2023-300897)
Supplement: Supplementary data [file bmjment-2023-300897supp001.pdf]

Online supplemental file for

Tracing Tomorrow: Young people's preferences and values related to use of personal sensing to predict mental health, using a digital game methodology

Gabriela Pavarini, David M· Lyreskog, Danielle Newby, Jessica Lorimer, Vanessa Bennett, Edward Jacobs, Laura Winchester, Alejo J Nevado-Holgado, Ilina Singh

This file includes:

Figure S1. Screenshots from Tracing Tomorrow: user receives letter stating they are at risk for depression (A); user chooses whether to find more information about it (B); brief text is displayed on screen outlining what happened next (C)
Table S1. Information about mental health and digital phenotyping Tracing Tomorrow users can unlock via clickable "fact" icons
Figure S2. Plot showing the overall dropout rate for 16–18-year-olds from questions 1-11
Table S2. The dropout rate from questions 1-11 stratified by either gender, age or country of origin
Figure S3. Odds ratio for differences in responses between females and other gender groups (male, other, did not say) regarding disclosure target preference (A) and willingness to disclose in peer group (B), controlling for age and nation of residence.
Figure S4. Odds ratio for differences in responses between females and other gender groups (male, other, did not say) regarding information- and help-seeking preferences following disclosure of mental health risk status**,** controlling for age and nation of residence.
Figure S5. Odds ratio for differences in responses between females and other gender groups (male, other, did not say) regarding normative disposition with regards to digital phenotyping services performed by school (A), social media platform (B) and data sharing between schools and health services (C)**,** controlling for age and nation of residence.
Figure S6. Odds ratio for differences in responses between females and other gender groups (male, other, did not say) regarding perceived impact of mental health risk information on self-understanding in the academic competence domain (A) and the moral domain (B).
Table S3. Summary of Question answers grouped by question theme and stratified by gender - Q2,9,10 (Information and support), Q3,5 (Trust to Disclose), Q4,6,11 (Normative disposition), Q7,8 (Self-understanding)
Table S4. Multinomial regression of gender effects to the questions from the Tracing Tomorrow game in adolescents aged 16-18, with the reference group as female – Q3,5 (Trust to disclose), Q7,8 (Self-understanding), Q2,9,10 (Information and Support), Q4,6,11 (Normative disposition)

Pavarini G, *et al. BMJ Ment Health* 2024; 27:e300897. doi: 10.1136/bmjment-2023-300897

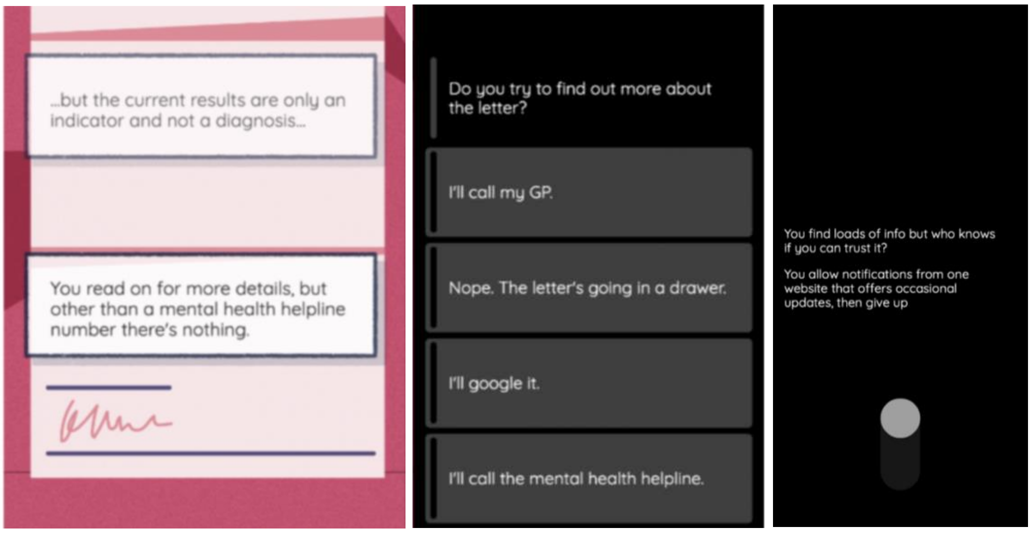

A B C

Figure S1. Screenshots from Tracing Tomorrow: user receives letter stating they are at risk for depression (A); user chooses whether to find more information about it (B); brief text is displayed on screen outlining what happened next (C)

Table S1. Information about mental health and digital phenotyping Tracing Tomorrow users can unlock via clickable "fact" icons

Fact 0	Tap on INFO ICONS to gather more information throughout the game. Tap again to close.
Fact 1	Internet and mobile phone tech can be used to collect or monitor signs of mental wellbeing in someone.
Fact 2	Depression can happen when anxiety about school or exams becomes common and too hard to cope with. Professional support could help.
Fact 3	Googling, posting on social media, texting, and messaging, are useful to services that monitor mental health risk. But allowing access to this data increases other risks, such as your privacy.
Fact 4	Risk predictions are just probabilities: having a high risk doesn't mean you'll definitely have mental health issues, and a low risk doesn't mean you definitely won't.
Fact 5	Schools could collect student data to help improve mental health risk profiles, but this could have other consequences. Students could experience stigma around their mental health.
Fact 6	Enough sleep, eating healthily and exercising can all help your mental health and wellbeing.

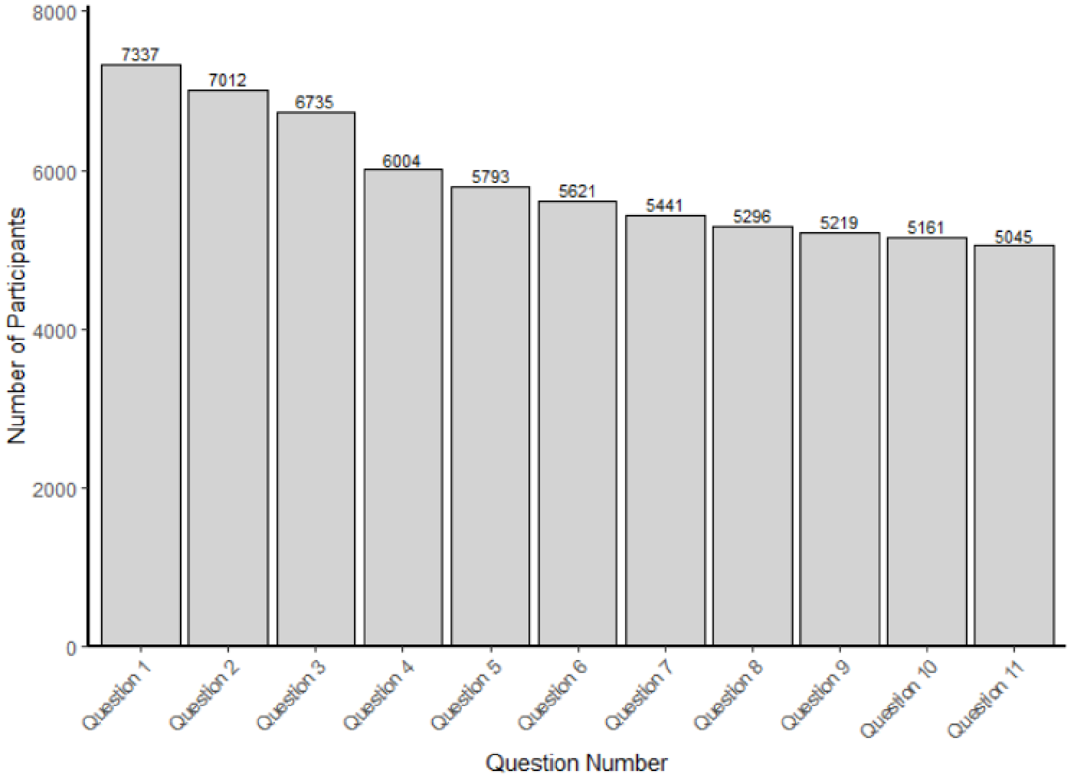

Figure S2. Plot showing the overall dropout rate for 16–18-year-olds from questions 1-11

Table S2. The dropout rate from questions 1-11 stratified by either gender, age or nation of origin

Group	Sub group	Dropout rate (%)
Gender	Did not say	36·99
	Female	29·09
	Male	37·19
	Other	23·3
Age	16	32·94
	17	31·15
	18	28·18
Nation	England	31·29
	Northern Ireland	31·24
	Scotland	29·88
	Wales	30·91

Gender differences

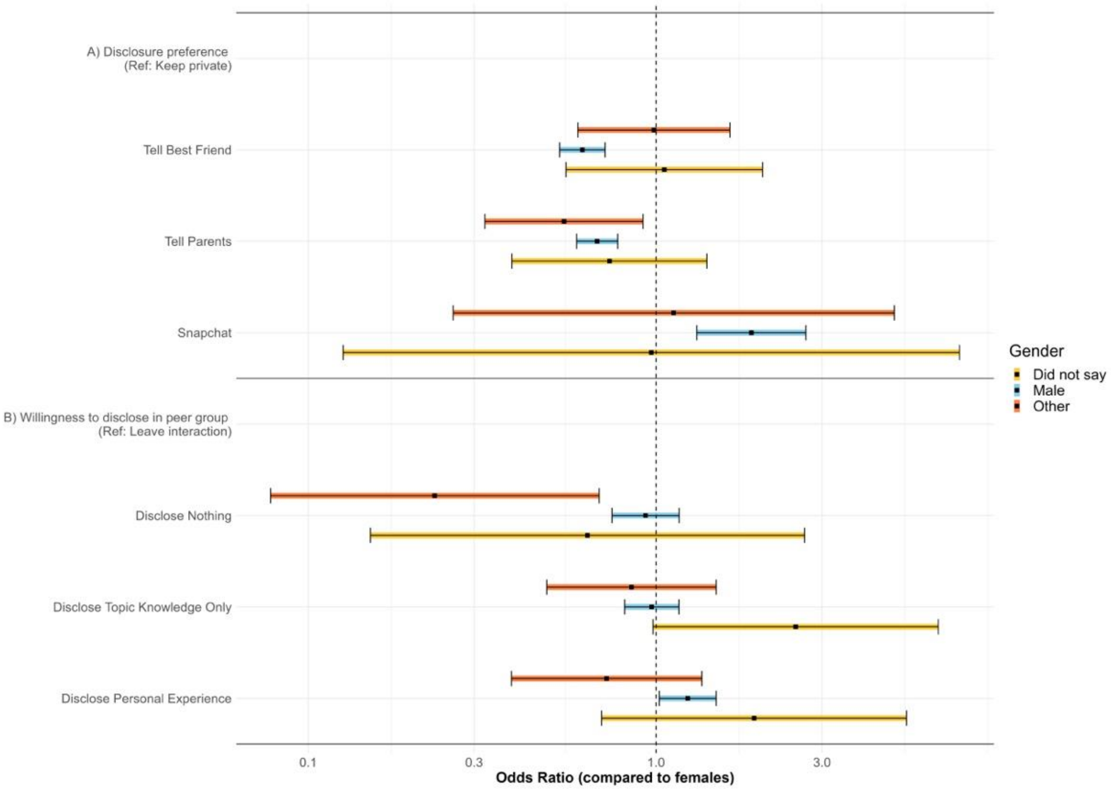

Figure S3. Odds ratio for differences in responses between females and other gender groups (male, other, did not say) regarding disclosure target preference (A) and willingness to disclose in peer group (B), controlling for age and nation of residence.

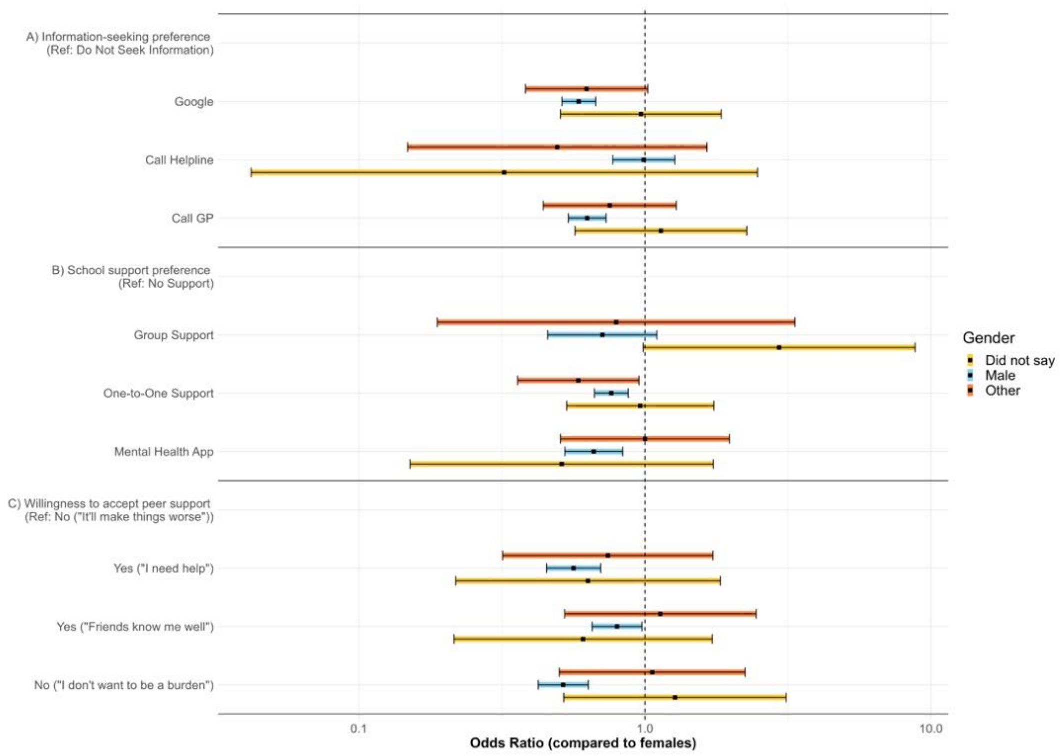

Figure S4. Odds ratio for differences in responses between females and other gender groups (male, other, did not say) regarding information- and help-seeking preferences following disclosure of mental health risk status**,** controlling for age and nation of residence.

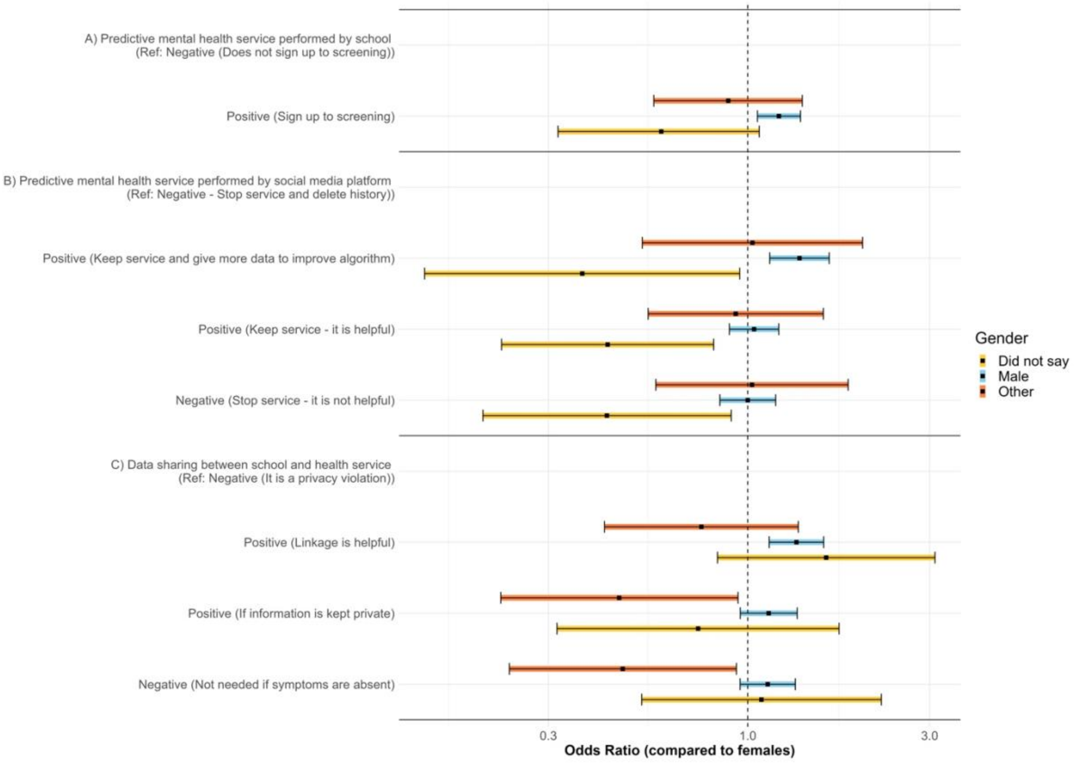

Figure S5. Odds ratio for differences in responses between females and other gender groups (male, other, did not say) regarding normative disposition with regards to digital phenotyping services performed by school (A), social media platform (B) and data sharing between schools and health services (C)**,** controlling for age and nation of residence.

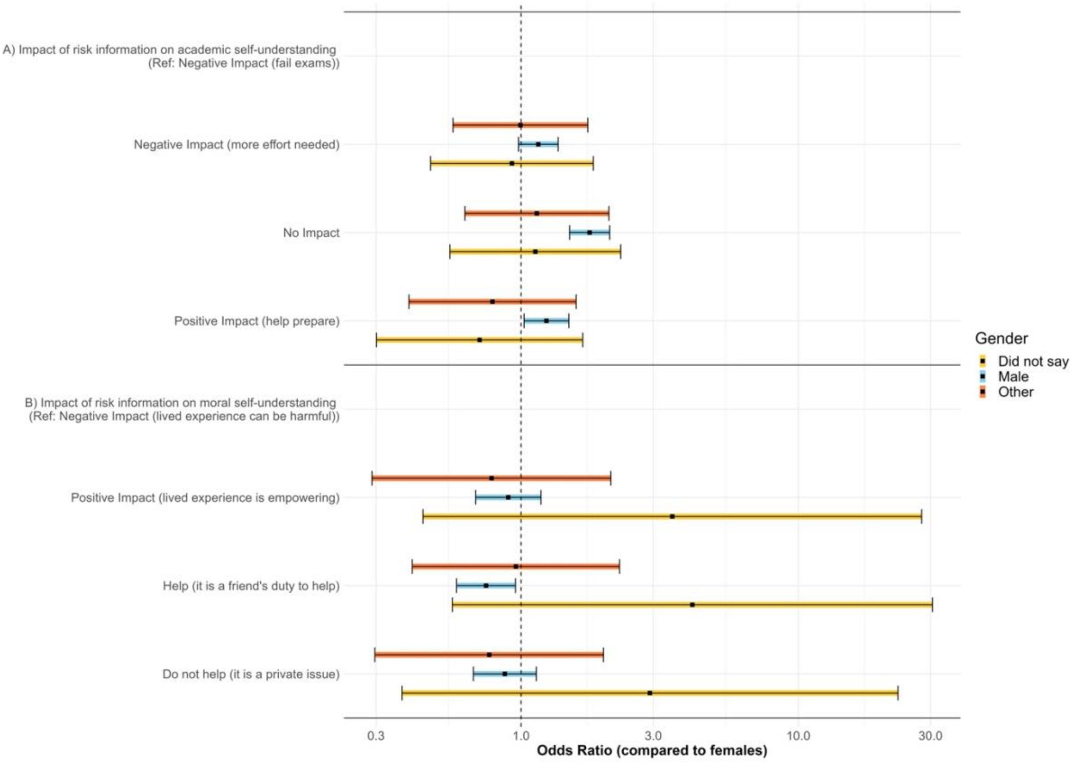

Figure S6. Odds ratio for differences in responses between females and other gender groups (male, other, did not say) regarding perceived impact of mental health risk information on self-understanding in the academic competence domain (A) and the moral domain (B).

Table S3. Summary of question answers grouped by question theme and stratified by gender - Q2,9,10 (Information and support), Q3,5 (Trust to Disclose), Q4,6,11 (Normative disposition), Q7,8 (Self-understanding)

Question reference category	Female (n = 4984)	Male (n = 1858)	Other (n = 101)	Did Not Say (n = 69)	All	p value	N
**Information and Support**							
Information-seeking preference (Q2)							
Google	2466 (49·5%)	773 (41·6%)	43 (42·6%)	33 (47·8%)	3315 (47·3%)	<0·001	7012
Call Helpline	218 (4·37%)	118 (6·35%)	3 (2·97%)	1 (1·45%)	340 (4·85%)		
Call GP	1372 (27·5%)	467 (25·1%)	29 (28·7%)	22 (31·9%)	1890 (27·0%)		
Do Not Seek Information	928 (18·6%)	500 (26·9%)	26 (25·7%)	13 (18·8%)	1467 (20·9%)		
School support preference (Q9)							
Group support	94 (2·46%)	27 (2·13%)	2 (2·50%)	4 (7·69%)	127 (2·43%)	<0·001	5219
One-to-One Support	1873 (49·0%)	561 (44·2%)	29 (36·2%)	25 (48·1%)	2488 (47·7%)		
Mental Health App	411 (10·8%)	109 (8·60%)	11 (13·8%)	3 (5·77%)	534 (10·2%)		
No Support	1441 (37·7%)	571 (45·0%)	38 (47·5%)	20 (38·5%)	2070 (39·7%)		
Willingness to accept peer support (Q10)							
Yes (“I need help”)	891 (23·6%)	251 (20·0%)	14 (17·5%)	8 (16·3%)	1164 (22·6%)	<0·001	5161
Yes (“Friends know me well”)	1046 (27·7%)	418 (33·4%)	25 (31·2%)	9 (18·4%)	1498 (29·0%)		
No (“I don’t want to be a burden”)	1421 (37·6%)	372 (29·7%)	32 (40·0%)	26 (53·1%)	1851 (35·9%)		
No (“It will make things worse”)	421 (11·1%)	212 (16·9%)	9 (11·2%)	6 (12·2%)	648 (12·6%)		
**Trust to disclose**							
Disclosure preference (Q3)							
Tell Best Friend	1518 (31·6%)	460 (26·1%)	39 (39·4%)	24 (36·9%)	2041 (30·3%)	<0·001	6735
Tell Parents	2234 (46·5%)	750 (42·6%)	32 (32·3%)	25 (38·5%)	3041 (45·2%)		
Snapchat	68 (1·41%)	63 (3·58%)	2 (2·02%)	1 (1·54%)	134 (1·99%)		
Keep private	989 (20·6%)	489 (27·8%)	26 (26·3%)	15 (23·1%)	1519 (22·6%)		
Willingness to disclose in peer group (Q5)							
Disclose Nothing	637 (15·2%)	195 (13·5%)	4 (4·65%)	3 (5·17%)	839 (14·5%)	<0·001	5793
Disclose Topic Knowledge Only	1793 (42·7%)	575 (39·8%)	42 (48·8%)	34 (58·6%)	2444 (42·2%)		
Disclose Personal Experience	1118 (26·6%)	456 (31·5%)	22 (25·6%)	16 (27·6%)	1612 (27·8%)		
Leave Interaction	655 (15·6%)	220 (15·2%)	18 (20·9%)	5 (8·62%)	898 (15·5%)		

**Normative disposition**							
Predictive mental health service performed by social media platform (Q4)							
Positive (Keep service and give more data to improve algorithm)	608 (14·0%)	269 (17·9%)	13 (14·6%)	5 (8·62%)	895 (14·9%)	0·002	6004
Positive (Keep service – it is helpful)	1448 (33·2%)	488 (32·6%)	28 (31·5%)	14 (24·1%)	1978 (32·9%)		
Negative (Stop service – it is not helpful)	932 (21·4%)	303 (20·2%)	20 (22·5%)	9 (15·5%)	1264 (21·1%)		
Negative (Stop service and delete history)	1370 (31·4%)	439 (29·3%)	28 (31·5%)	30 (51·7%)	1867 (31·1%)		
Data sharing between school and health service (Q6)							
Positive (Linkage is helpful)	739 (18·1%)	302 (21·7%)	15 (18·1%)	15 (26·8%)	1071 (19·1%)	0·003	5621
Positive (if information is kept private)	728 (17·8%)	256 (18·4%)	9 (10·8%)	7 (12·5%)	1000 (17·8%)		
Negative (Not needed if symptoms are absent)	798 (19·5%)	277 (19·9%)	10 (12·0%)	11 (19·6%)	1096 (19·5%)		
Negative (It is a privacy violation)	1828 (44·7%)	554 (39·9%)	49 (59·0%)	23 (41·1%)	2454 (43·7%)		
Predictive mental health service performed by school (Q11)							
Positive (Sign up to screening)	1842 (49·8%)	666 (54·5%)	37 (46·8%)	17 (37·0%)	2562 (50·8%)	0·007	5045
Negative (Does not sign up to screening)	1855 (50·2%)	557 (45·5%)	42 (53·2%)	29 (63·0%)	2483 (49·2%)		
**Impact of risk information on self-understanding**							
Academic (Q7)							
Negative impact (fail exams)	1527 (38·4%)	419 (31·4%)	31 (38·8%)	22 (40·0%)	1999 (36·7%)	<0·001	5441
Negative Impact (more effort needed)	1034 (26·0%)	329 (24·7%)	21 (26·2%)	14 (25·5%)	1398 (25·7%)		
No Impact	732 (18·4%)	356 (26·7%)	17 (21·2%)	12 (21·8%)	1117 (20·5%)		
Positive Impact (help prepare)	680 (17·1%)	229 (17·2%)	11 (13·8%)	7 (12·7%)	927 (17·0%)		
Moral (Q8)							
Positive Impact (lived experience is empowering)	660 (17·0%)	242 (18·8%)	12 (15·0%)	9 (17·0%)	923 (17·4%)	0·106	5296
Help (it is a friend's duty to help)	2065 (53·3%)	627 (48·6%)	46 (57·5%)	33 (62·3%)	2771 (52·3%)		
Negative Impact (lived experience can be harmful)	256 (6·61%)	105 (8·14%)	6 (7·50%)	1 (1·89%)	368 (6·95%)		
Do not help (privacy)	892 (23·0%)	316 (24·5%)	16 (20·0%)	10 (18·9%)	1234 (23·3%)		

Table S4. Multinomial regression of gender effects to the questions from the Tracing Tomorrow game in adolescents aged 16-18, with the reference group as female – Q3,5 (Trust to disclose), Q7,8 (Self-understanding), Q2,9,10 (Information and Support), Q4,6,11 (Normative disposition)

Question reference category	Category	Male			Other			Did Not Say		
		OR	95%CI	p-value	OR	95%CI	p-value	OR	95%CI	p-value
**Trust to disclose**										
Disclosure preference (Q3) (Ref: Keep private)	Best Friend	0·614	0·528-0·714	**< 0·001**	0·986	0·596-1·631	0·957	1·056	0·551-2·024	0·869
	Parents	0·677	0·591-0·776	**< 0·001**	0·544	0·322-0·917	**0·022**	0·735	0·385-1·4	0·349
	Snapchat	1·879	1·309-2·695	**0·001**	1·123	0·261-4·838	0·876	0·969	0·126-7·454	0·976
Willingness to disclose in peer group (Q5) (Ref: Leave interaction)	Disclose Nothing	0·933	0·747-1·165	0·539	0·231	0·078-0·686	**0·008**	0·635	0·151-2·672	0·536
	Disclose Topic Knowledge Only	0·971	0·812-1·163	0·752	0·85	0·485-1·489	0·571	2·519	0·98-6·475	0·055
	Full Disclosure	1·233	1·022-1·489	**0·029**	0·721	0·384-1·355	0·309	1·914	0·697-5·251	0·208
**Impact of risk information on self-understanding**										
Academic (Q7) (Ref: Negative impact (fail exams))	Negative Impact (more effort needed)	1·154	0·979-1·361	0·088	0·995	0·568-1·741	0·985	0·927	0·472-1·822	0·827
	No Impact	1·767	1·496-2·088	**< 0·001**	1·14	0·627-2·075	0·667	1·126	0·554-2·288	0·743
	Positive Impact (help prepare)	1·235	1·026-1·487	**0·025**	0·789	0·394-1·58	0·504	0·709	0·301-1·669	0·431
Moral (Q8) (Ref: Do not help (Negative experience can be harmful))	Positive Impact (lived experience is empowering)	0·899	0·686-1·18	0·443	0·783	0·29-2·108	0·628	3·512	0·443-27·858	0·235
	Help (it is a friend's duty to help)	0·748	0·585-0·955	**0·02**	0·958	0·405-2·265	0·921	4·149	0·565-30·467	0·162
	Do not help (privacy)	0·874	0·673-1·135	0·312	0·768	0·297-1·983	0·585	2·916	0·372-22·888	0·309

**Information and support**										
Information-seeking preference (Q2) (Ref: Do Not Seek Information)	Google	0·587	0·513-0·673	**< 0·001**	0·625	0·382-1·024	0·062	0·969	0·507-1·85	0·923
	Call Helpline	0·991	0·772-1·273	0·945	0·494	0·148-1·648	0·251	0·322	0·042-2·476	0·276
	Call GP	0·628	0·54-0·731	**< 0·001**	0·753	0·441-1·287	0·3	1·138	0·57-2·272	0·713
School support preference (Q9) (Ref: No support)	Group Support	0·71	0·457-1·101	0·126	0·794	0·188-3·345	0·753	2·947	0·986-8·813	0·053
	One-to-one Support	0·763	0·666-0·874	**< 0·001**	0·585	0·359-0·953	**0·031**	0·963	0·533-1·742	0·902
	Mental Health App	0·662	0·525-0·836	**0·001**	1·001	0·507-1·976	0·998	0·512	0·151-1·734	0·282
Willingness to accept peer support (Q10) (Ref: No ("It will make things worse"))	Yes ("I need help")	0·563	0·453-0·7	**< 0·001**	0·742	0·318-1·729	0·489	0·632	0·218-1·836	0·399
	Yes ("Friends know me well")	0·799	0·654-0·977	**0·029**	1·133	0·524-2·45	0·751	0·608	0·215-1·721	0·349
	No ("I don't want to be a burden")	0·518	0·424-0·634	**< 0·001**	1·061	0·502-2·242	0·877	1·274	0·52-3·119	0·596
**Normative disposition**										
Predictive service by social media platform (Q4) (Ref: Negative (Stop service and delete history))	Positive (Keep service and give more data to improve algorithm)	1·367	1·142-1·636	**0·001**	1·029	0·529-2·001	0·933	0·368	0·142-0·953	**0·04**
	Positive (Keep service – it is helpful)	1·039	0·895-1·206	0·617	0·93	0·548-1·579	0·788	0·429	0·226-0·814	**0·01**
	Negative (Stop service – it is not helpful)	1	0·845-1·184	1	1·026	0·574-1·834	0·93	0·427	0·202-0·905	**0·026**
Data sharing between school and health service (Q6) (Ref: Negative (It is a privacy violation))	Positive (Linkage is helpful)	1·342	1·138-1·582	**< 0·001**	0·756	0·421-1·357	0·349	1·606	0·833-3·098	0·157
	Positive (if information is kept private)	1·135	0·956-1·348	0·148	0·46	0·225-0·943	**0·034**	0·741	0·316-1·736	0·49
	Negative (Not needed if symptoms are absent)	1·128	0·955-1·333	0·157	0·47	0·237-0·934	**0·031**	1·086	0·527-2·241	0·823
Predictive mental health service performed by school (Q11) (Ref: Negative (Does not sign up to screening))	Positive (Sign up to screening)	1·207	1·06-1·375	**0·004**	0·889	0·567-1·39	0·607	0·593	0·318-1·072	0·089

p values < 0·05 are in bold
